# The Burden of Peripheral Artery Disease in China From 1990 to 2019 and Forecasts for 2030: Findings From the Global Burden of Disease Study 2019

**DOI:** 10.3389/ijph.2024.1607352

**Published:** 2024-12-17

**Authors:** Ye Hu, Jiyue Gao, Qiping Zhuo, Huixin Liu, Meiling Wang, Nina Jiang, Xueqing Wang, Kainan Wang, Zuowei Zhao, Man Li

**Affiliations:** ^1^ Department of Oncology, The Second Hospital of Dalian Medical University, Dalian, Liaoning, China; ^2^ Department of Breast Surgery, The Second Hospital of Dalian Medical University, Dalian, Liaoning, China; ^3^ Department of Science and Education, Dalian Municipal Central Hospital, Dalian, Liaoning, China

**Keywords:** death rate, incidence, disability-adjusted life years, China, peripheral artery disease

## Abstract

**Objectives:**

The incidence of peripheral arterial disease (PAD) in China is increasing. We aim to conduct a comprehensive analysis of the burden of PAD.

**Methods:**

We collected information from 1990 to 2019 in the Global Burden of Disease (GBD 2019) study. Joinpoint regression analysis was used to calculate the annual percentage change (APC). Trends in incidence, mortality and DALYs were forecasted by Bayesian age-period-cohort (BAPC) analysis.

**Results:**

In 2019, the number of new cases and prevalence of PAD in China accounted for nearly a quarter of the global proportion. The age-standardized incidence rate (ASIR) declined after rising until 2005. The age-standardized death rate (ASDR) maintained an upward trend. The DALYs was 0.16 million. Incidence, prevalence and DALYs are predominantly female, except for mortality, which is predominantly male. Smoking predominantly affected males, while hypertension and diabetes had a greater impact on females. By 2030, ASDR is elevated, predominantly in males. ASIR and age-standardized DALY rate decline, predominantly in females.

**Conclusion:**

It is urgent for China to develop strategies based on the specific distribution characteristics of the PAD burden.

## Introduction

Peripheral arterial disease (PAD) is defined as the signs and symptoms of ischemia due to narrowing or blockage of peripheral arteries caused by atherosclerosis. And PAD is most often found in the arteries of the lower limbs, with the main symptoms being intermittent claudication and resting pain. PAD has a strong covariance with cardiovascular disease and is an important guide to cardiovascular risk [1]. PAD has been reported to lead to a 60% increased risk of all-cause mortality, a 96% increased risk of cardiovascular death, a 45% increased risk of coronary artery disease and a 35% increased risk of cerebrovascular disease [2]. However, PAD receives less attention far less than coronary heart disease or stroke [3].

According to the Global Burden of Disease 2019 study, there are over 113 million people with PAD worldwide. PAD negatively impacts health-related living and treatment, resulting in 1.54 million disability-adjusted life years (DALY) in 2019 globally [4]. A study reviewing hospitalizations of patients with PAD in the US in 2014 found that the hospitalization rate for PAD was 89.5 per 100,000 [5]. Thus, PAD can place a significant medical and economic burden on society and the healthcare system.

The burden of atherosclerotic cardiovascular disease is rapidly shifting from high-income to middle- and low-income countries [6]. A recent study published data on global PAD prevalence trends from 2000 to 2010, during which the number of people with PAD increased by 28.7% in low- and middle-income countries and by 13.1% in high-income countries [7]. Thus, the prevalence of PAD is currently higher in middle and low-income countries. China is the largest developing country in the world and currently has a serious population ageing problem [8]. Meanwhile, China is the world’s largest consumer of tobacco, with over 300 million adult smokers [9]. These are important risk factors for PAD [6], and there is an important need for an epidemiological investigation study to analyze the burden of PAD in China.

The Global Burden of Disease (GBD) study is a comprehensive assessment of burden metrics due to disease, injury and risk factors through publicly available databases [10]. Previously, a few studies have analyzed the epidemiological trends of PAD at global and national levels using data from GBD 2010 [11] and GBD 2019 [12], but few studies have been conducted on the burden of PAD in China. Assessing the burden of disease for PAD in China is of great significance to fill this data gap and provide a basis for changing the current situation of low public awareness of PAD [13]. In this study, we assessed the burden of PAD in China from 1990 to 2019, using incidence, death rate and DALYs as measures and analyzing population characteristics such as age, sex, and risk factors. We also conducted a predictive analysis of the burden of PAD in China over the next 10 years. It aims to provide important information for the planning of public health services for PAD, and to provide a reference for future policy design and health resource allocation.

## Methods

### Data Source

Design protocols and standardized processes for global burden of disease (GBD) studies have been widely reported in many relevant studies [10, 14, 15]. The GBD database (https://vizhub.healthdata.org/gbd-results-tool/) provides comprehensive data for 204 countries and territories with 369 causes and associated 87 risk factors for the period 1990 to 2019. In the list of selected causes of GBD, PAD is a level 3 independent and non-disaggregated cause under the first level of non-communicable diseases and the second level of cardiovascular diseases. The diagnosis of PAD was determined according to the codes of the International Classification of Diseases (ICD), 10th Revision [16]. In this study, the ICD-10 codes for PAD diagnosis included I70.2 and I73.9.

### Disease Burden Metrics

We obtained the prevalence and number of deaths from PAD through the GBD database, representing the number of new cases of PAD and the number of deaths due to PAD in the selected period, respectively. The prevalence was also collected for the proportion of people in the population who developed PAD and related injuries or sequelae. DALYs can be viewed as years of health lost because of disability and include both years lived with disability (YLDs) and years of life lost due to premature mortality (YLLs), while the value of DALYs is equal to the sum of the two. The age-standardized rates (ASR, per 100,000 population) were calculated to eliminate differences in the age distribution of each evaluation indicator.

### Attributable Risk Factors for PAD

Data on PAD-related risk factors and population characteristics, time and region were obtained in the GBD database. A total of 7 potential risk factors were screened after considering adequate causal evidence, availability of exposure data, including smoking, high systolic blood pressure (SBP), high fasting plasma glucose, kidney dysfunction, diet high in sodium, lead exposure, and environmental or occupational risks. A comprehensive assessment of the impact of attributed risk factors on ASDRs and DALYs was conducted using the previously reported research methods [15].

### Temporal Trends and Prediction Analysis of PAD Burden China

Trends in PAD burden from 1990 to 2019 are assessed with Joinpoint software (version 4.9.1.0, April 2023; Statistical Research and Applications Branch, National Cancer Institute). Joinpoint regression analysis uses a log-linear model (ln y = xb) for linear segmentation of the time line to estimate trends in one or more line segments representing a given time horizon. Monte Carlo permutation tests were used for model preference to determine the nodes of change in the data, taking into account the minimum error and the optimal number of nodes. The internal trend of each independent interval is evaluated by calculating the annual percentage change (APC) of the segmented ASDR and ASIR for each year. The average APC (AAPC) reflecting the trend of the entire interval from 1990 to 2019 was calculated by geometrically weighting the regression coefficients of each interval on the basis of the APC.

The INLA package (23.04.24 version), BAPC package (0.0.36 version) and easyGBD package (1.0.0.3 version) were downloaded in R software (4.2.3 version, The R Foundation for Statistical Computing), and the Bayesian age-period-cohort (BAPC) model method was used to predict the future PAD burden in China. The BAPC model is a Bayesian model added to the age-period-cohort analysis model can solve the difficulties in parameter estimation due to the linear relationship between the 3 factors in the age-period-cohort model. Age, period, and/or cohort effects can be optimized by a random walk of second order (RW2) construction models for more accurate prediction of future incidence, number of deaths, ASIR, and ASDR.

### Statistical Analysis

Estimated annual percentage change (EAPC) is used to show the time trend of each age-specific standardized rate burden indicator. The EAPC is calculated using a linear regression equation, where the y value is the corresponding ln (ASR) for each year and the x value is the year. The EAPC = 100*[exp(β)-1] was obtained by taking the corresponding x and y values and calculating the β value based on the regression equation. EAPC >0 means that ASR is increasing from year to year; EAPC <0 means that ASR is decreasing from year to year. We provide 95% uncertainty intervals (UIs) for each disease burden indicator, calculated by taking the 25th and 975th ordered quantities from the posterior distribution of 1,000 samples from each data modeling process [10]. The UIs were obtained using correlation matrices for multiple replicate sampling calculations, taking into account the differences between countries and different calculation methods, as well as the presence of uncertainty in missing data values for multiple filling. Statistically significant was defined as a *p*-value less than 0.05. The data in this paper were visualized by GraphPad Prism (9.0 version, GraphPad Software, Inc., United States) and R software.

## Results

### Overall Findings About Disease Burden Metrics Caused by PAD

As shown in Table 1, the burden of PAD in China in 1990 and 2019 was measured by incidence, prevalence, number of deaths, DALYs, YLDs and YLLs, as well as the corresponding ASRs and trends. The number of new incidences of PAD in China reached 2.62 million (95% UI: 2.25–3.01) in 2019, an increase of 154.07% compared to 1.03 million (95% UI: 0.89–1.18) new cases in 1990. The number of prevalent cases of PAD increased by 173.94%, from 10.40 million (95% UI: 8.92–11.89) in 1990 to 28.49 million (95% UI: 24.55–36.61) in 2019. The number of deaths in PAD increased by 262.73% to 2.21 thousands (95% UI: 1.81–2.74) in 2019 compared to 1990. The number of DALYs in 2019 was 165.73 thousands (95% UI: 96.08–274.01) also increased 145.27% from 67.57 thousands (95% UI: 37.81–110.28) in 1990. Meanwhile, the YLDs and YLLs of Chinese PAD patients increased from 55.80 (95% UI: 25.80–98.81) and 11.77 (95% UI: 9.29–16.19) in 1990 to 130.06 (95% UI: 59.47–234.46) and 35.67 (95% UI: 29.28–43.40) in 2019, respectively.

**TABLE 1:** The number and age-standardized rates of incidence, prevalence, deaths, disability-adjusted life years, years lived with disability, and years of life lost, and their temporal trends (China, 1990–2019).

	1990		2019		1990–2019
	Number [10^3^ (95% UI)]	ASR [per 100,000 (95% UI)]	Number [10^3^ (95% UI)]	ASR [per 100,000 (95% UI)]	EAPC of ASR [% (95% CI)]
Incidence	1029.59 (887.38, 1177.95)	121.45 (105.55, 138.63)	2615.88 (2251.15, 3008.61)	125.43 (109.07, 143.36)	0.02 (−0.05, 0.1)
Prevalence	10399.94 (8919.42, 11886.89)	1330.42 (1153.94, 1516.87)	28489.64 (24548.56, 32612.50)	1423.78 (1234.84, 1625.31)	0.16 (0.08, 0.23)
Deaths	0.61 (0.48, 0.83)	0.12 (0.09, 0.16)	2.21 (1.81, 2.74)	0.14 (0.11, 0.18)	0.62 (0.54–0.7)
DALYs	67.57 (37.81, 110.28)	9.65 (5.41, 15.99)	165.73 (96.08, 274.01)	8.82 (5.14, 14.4)	−0.45 (−0.52, −0.39)
YLDs	55.80 (25.80, 98.81)	7.95 (3.74, 14.19)	130.06 (59.47, 234.46)	6.87 (3.19, 12.47)	−0.68 (−0.77, −0.59)
YLLs	11.77 (9.29, 16.19)	1.69 (1.32, 2.29)	35.67 (29.28, 43.40)	1.96 (1.60, 2.40)	0.5 (0.42, 0.57)

DALYs, Disability-Adjusted Life Years; YLDs, Years Lived with Disability; YLLs, Years of Life Lost.

**FIGURE 1:**
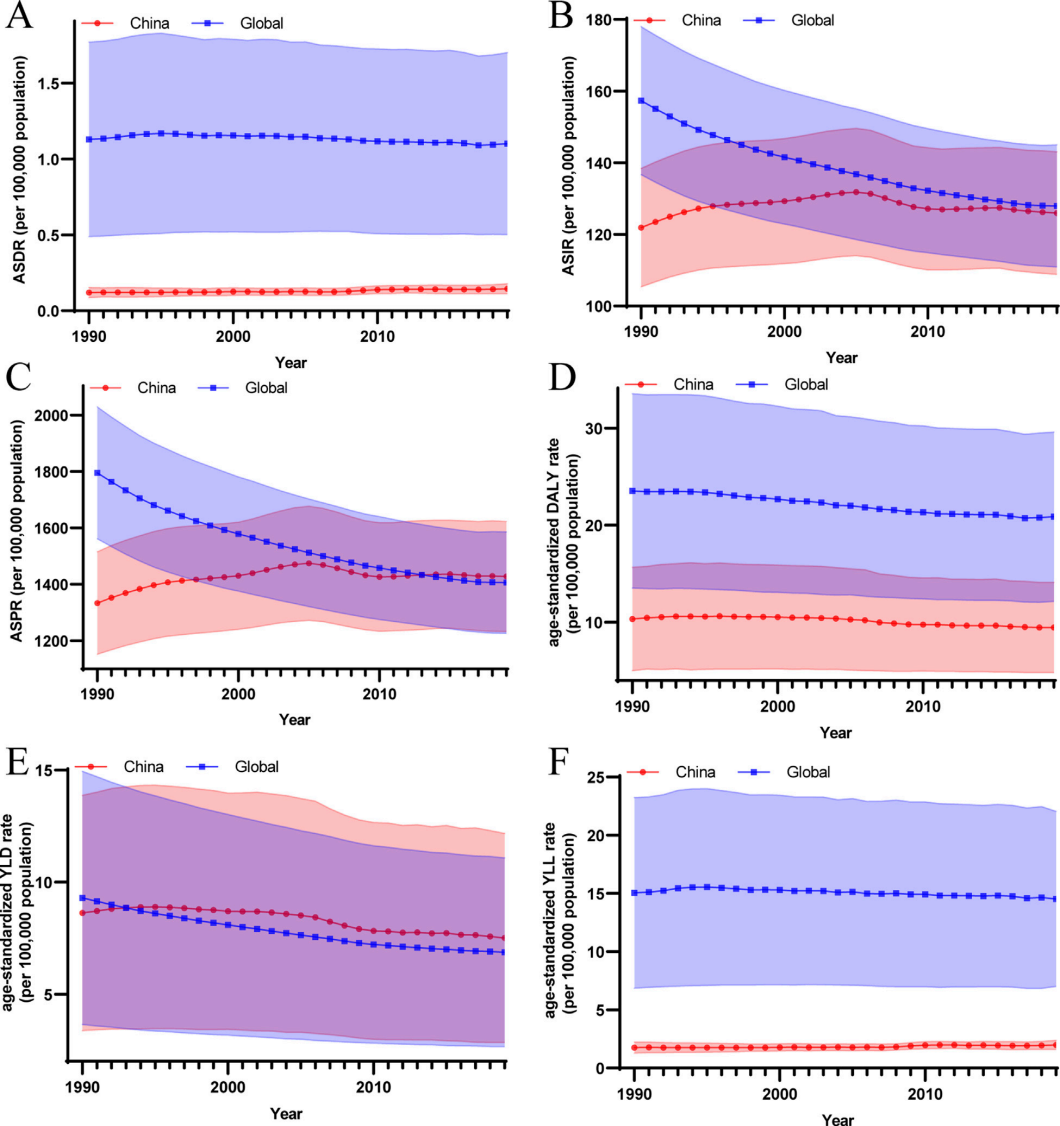
The temporal trend of the age-standardized rates for each disease burden metric caused by peripheral arterial disease (China and global, 1990–2019). **(A)** The temporal trend of age-standardized death rate; **(B)** The temporal trend of age-standardized incidence rate; **(C)** The temporal trend of age-standardized prevalence rate; **(D)** The temporal trend of age-standardized disability-adjusted life years rate; **(E)** The temporal trend of age-standardized years lived with disability rate; **(F)** The temporal trend of age-standardized years of life lost rate. ASIR, age-standardized incidence rate; ASPR, age-standardized prevalence rate; ASDR, age-standardized death rate; DALY, Disability-Adjusted Life Years; YLD, Years Lived with Disability; YLL, Years of Life Lost.

The ASRs for each metric of PAD burden in China changed marginally between 1990 and 2019 (Figure 1). In contrast, age-standardized DALY rate showed a downward trend from 9.65 (95% UI: 5.41–15.99) in 1990 to 8.82 (95% UI: 5.14–14.4) in 2019, with an EAPC being −0.45 (95% UI: −0.52 to −0.39). The PAD-related healthy life loss in China was dominated by YLD, which accounted for 82.58% and 78.48% of DALYs in 1990 and 2019, respectively. The age-standardized YLD rate decreased from 7.95 (95% UI: 3.74–14.19) in 1990 to 6.87 (95% UI: 3.19–12.47) in 2019, with an EAPC of −0.68 (95% UI: −0.77 to −0.59). And the age-standardized YLL rate for the burden of PAD in China maintained an upward trend from 1.69 (95% UI: 1.32–2.29) in 1990 to 1.96 (95% UI: 1.60–2.40), with an EAPC being 0.5 (95% UI: 0.42–0.57).

According to GBD 2019 data, from 1990 to 2019, the number of people with PAD in China rose from 15.81% to 25.11% of the total number of people with PAD worldwide. Compared to the mild increase in ASIR and ASPR of PAD burden in China from 1990 to 2019, the global ASIR and ASPR of PAD burden showed a significant decreasing trend. In 2019, the global ASIR decreased to 127.11 (95% UI: 111.28–145.44) slightly higher than that of China, while the ASPR decreased to 1401.85 (95% UI: 1228.48–1589.39) lower than that of China. In addition, the age-standardized DALY rate and YLL rate in the burden of PAD in China were both significantly lower than the global ones between 1990 and 2019, respectively. The age-standardized DALY rate and YLL rate of the global PAD burden in 2019 reached 19.55 (95% UI: 12.91–30.21) and 13.26 (95% UI: 7.71–22.61), respectively. In contrast, the global age-standardized YLD rate from 1990 to 2019 showed a decreasing trend and was close to the value of age-standardized YLD rate with China. The global age-standardized YLD rate in 2019 is lower than that of China at 6.29 (95% UI: 2.97–11.35) (Figure 1; Supplementary Table S1).

### Age-Specific Burden of PAD in Males and Females From 1990 to 2019

**FIGURE 2:**
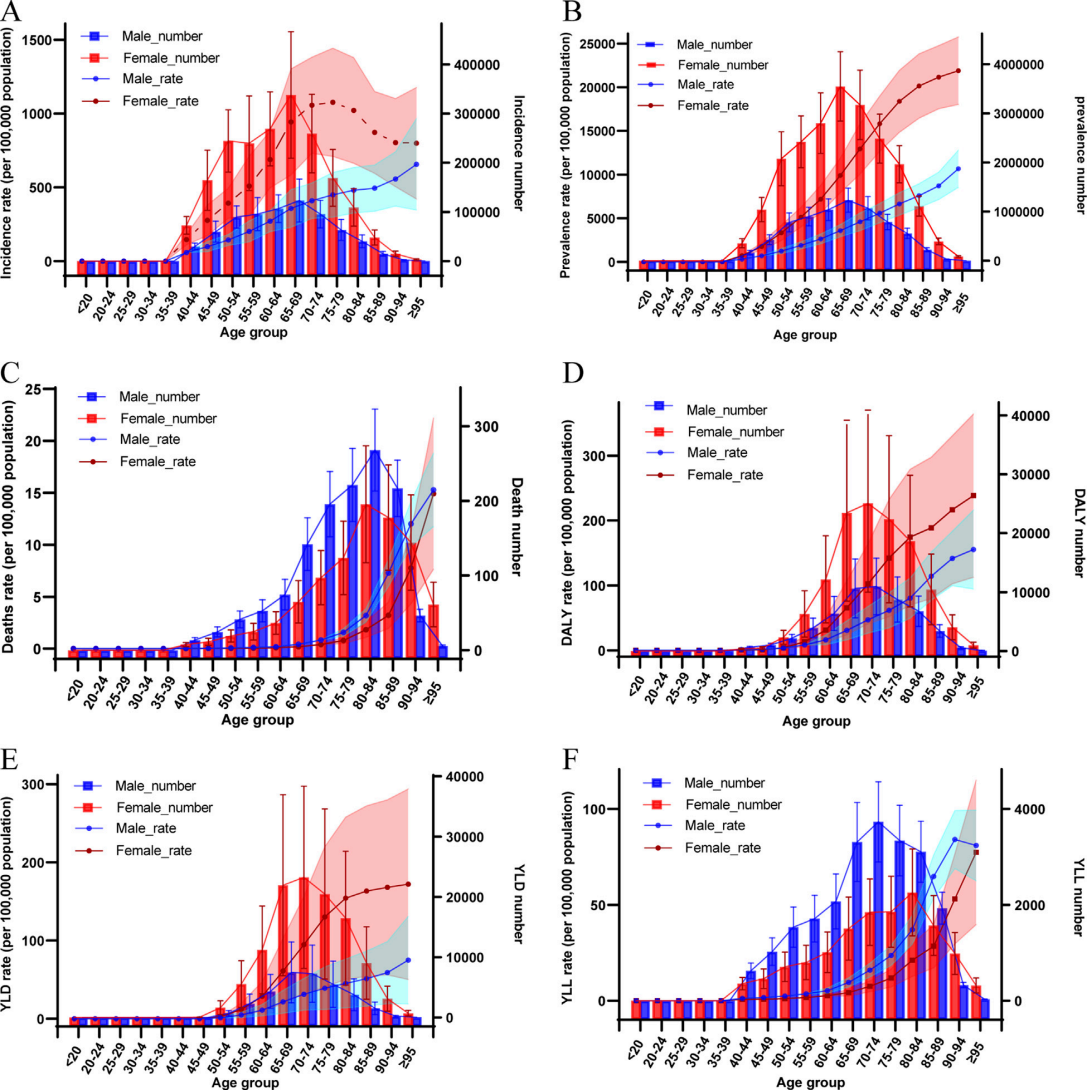
Comparison of the distribution of incidence **(A)**, prevalence **(B)**, death **(C)**, disability-adjusted life years **(D)**, years lived with disability **(E)** and years of life lost **(F)** due to peripheral arterial disease and corresponding crude rates of disease burden metrics by age group and sex (China, 2019). Shaded areas show 95% uncertainty intervals. DALY, Disability-Adjusted Life Years; YLD, Years Lived with Disability; YLL, Years of Life Lost.

In the GBD 2019 database, all Chinese PAD patients were over 40 years of age. The disease burden metrics caused by PAD in China increased significantly in all age groups compared to 1990, especially the metrics increased more and more as the age groups got older (Supplementary Table S2). In 2019, new cases of PAD in China were mainly concentrated between 45 and 79 years of age, with a maximum of 0.45 million (95% UI: 0.29–0.64) cases between 65 and 69 years of age, and far more women than men. The crude incidence rates of PAD per 100,000 population continued to increase with age in the male subgroup. And in the female subgroup the highest score [768.17 (95% UI: 522.01–1046.56)] was reached at the age of 75–79 years. In 2019, the distribution of PAD prevalence cases by age group in China was similar to that of incidence, while in terms of crude prevalence rates, both male and female subgroups maintained a continuous upward trend with increasing age. In 2019, the number of PAD deaths in China was concentrated between the ages of 65 and 95 years, peaking at 454 (95% UI: 369–563) among the ages of 80–84 years. The number of male deaths was higher than that of female in each age stratum until the age of 90 years, while the opposite was true after 90 years. The crude mortality rates of male PAD patients rose rapidly and then slowly with increasing age, whereas the opposite trend was observed for females. However, the subgroups of males and females had the highest values in the age group subgroup above 95 years, and both had close crude mortality rates. In addition, the DALYs, YLDs, YLLs of PAD burden in China in 2019 were mainly distributed between 65 and 84 years of age, and were highest at 70–74 years of age. Female patients had more DALY and YLD than male patients in all age strata. YLL was significantly higher in male patients than in female patients, but the PAD-related healthy life loss in male patients was still predominantly YLD, accounting for 57% of DALY (Figure 2).

### Attributable Risk Factors for PAD Burden From 1990 to 2019

**FIGURE 3:**
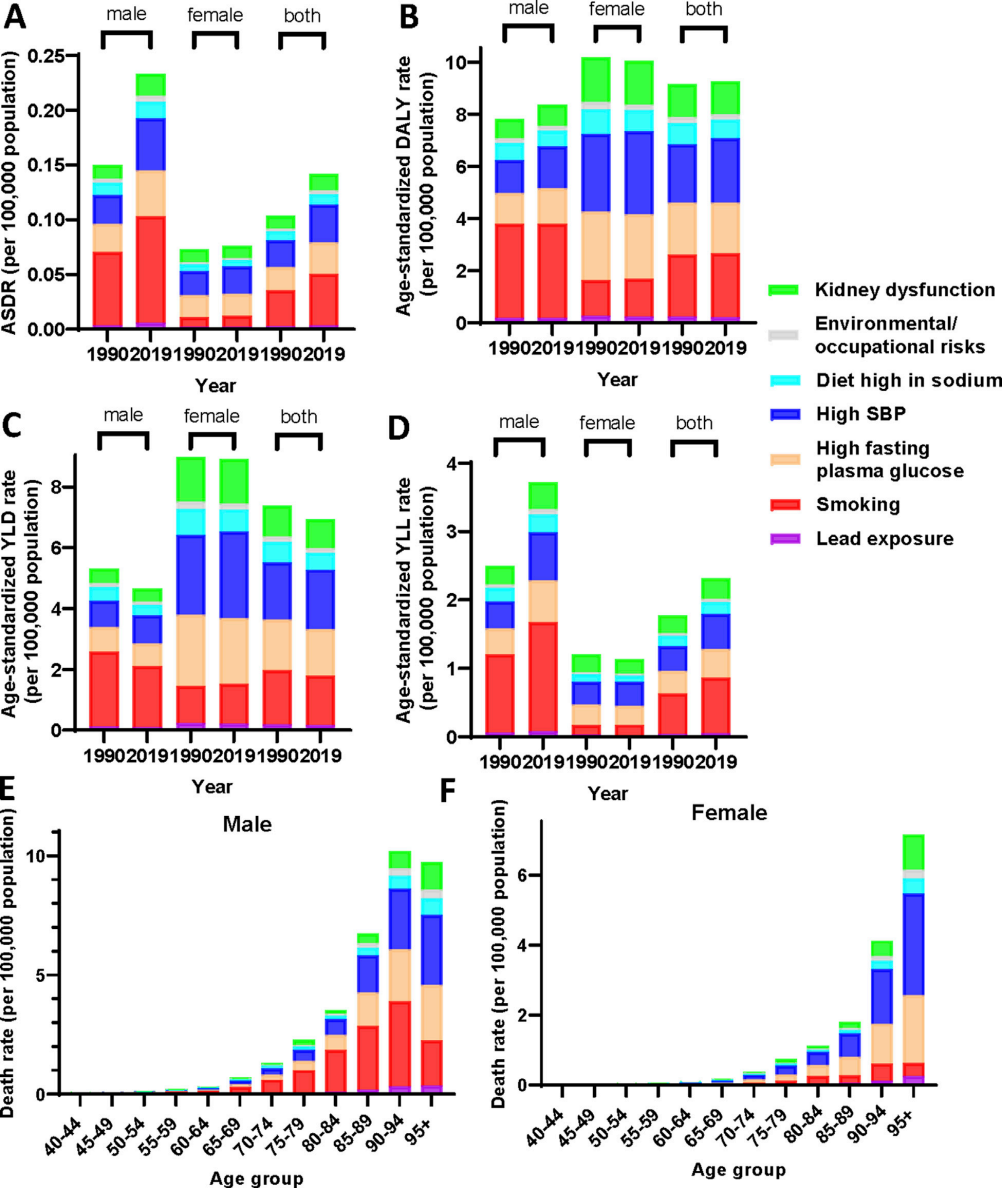
Distribution of attributable risk factors for peripheral arterial disease burden across gender subgroups and in age stratification (China, 1990–2019). Age-standardized death rate **(A)**, age-standardized disability-adjusted life years rate **(B)**, age-standardized years lived with disability rate **(C)** and age-standardized years of life lost rate **(D)** for peripheral arterial disease by risk factor and sex in 1990 and 2019. The attributed death rate of risk factors for peripheral arterial disease in 2019 in different age groups in male **(E)** and female **(F)**. ASDR, age-standardized death rate; DALY, Disability-Adjusted Life Years; YLD, Years Lived with Disability; YLL, Years of Life Lost; SBP, systolic blood pressure.

To further explore the composition of PAD burden, we explored disease-related attributable risk factors, screening for risk factors such as smoking, high systolic blood pressure (hypertension), high fasting plasma glucose (diabetes), kidney dysfunction, and diet high in sodium. Smoking was the most common risk factor for PAD burden in 2019, contributed to an ASDR per 100,000 of 0.047 (95% UI: 0.037–0.057) and 33.58% (95% UI: 27.32–39.48) of PAD-related death. And smoking in particular was the most prominent risk factor in the burden of PAD in male, contributed to an ASDR per 100,000 of 0.098 (95% UI: 0.078–0.118) and 49.22% (95% UI: 44.24–53.42) of PAD-related death, as well as an age-standardized DALY rate per 100,000 of 3.60 (95% UI: 2.41–5.27) and 59.94% (95% UI: 51.27–65.65) of PAD-related DALYs. Hypertension and diabetes mellitus are also major risk factors for PAD burden, and both are more predominant in female patients in particular. Hypertension as an attributable risk factor for PAD burden accounted for an ASDR per 100,000 of 0.035 (95% UI: 0.023–0.051) and 24.76% (95% UI: 17.28–33.44) of PAD-related death rate, as well as an age-standardized DALY rate per 100,000 of 2.46 (95% UI: 1.33–4.26) and 27.85% (95% UI: 20.35–35.35) of PAD-related DALYs. Similarly, diabetes also represented the most prominent risk factor for PAD burden in 2019, responsible for an ASDR per 100,000 of 0.029 (95% UI: 0.022–0.037) and 20.36% (95% UI: 17.35–23.68) of PAD-related death, as well as an age-standardized DALY rate per 100,000 of 1.95 (95% UI: 1.11–3.20) and 22.07% (95% UI: 19.41–24.92) of PAD-related DALYs (Figure 3).

From 1990 to 2019, the annual change in ASDR influenced by the major risk factors in female PAD patients was small. However, the ASDR due to these three main risk factors in male PAD patients maintained a yearly increasing trend and the increasing trend became more significant after 2008 (Supplementary Figure S1). Overall, the ASDR for PAD burden due to different risk factors was significantly higher in 2019 compared to 1990, with a smaller change in age-standardized DALY rates. However, compared to 1990, YLD and YLL due to different risk factors in 2019 showed a difference in decrease and increase, respectively. The YLD and YLL due to different risk factors decreased mildly in 2019 for female patients compared to 1990.

### Temporal Trends of PAD Burden by Sex and Age Groups From 1990 to 2019 in China

**FIGURE 4:**
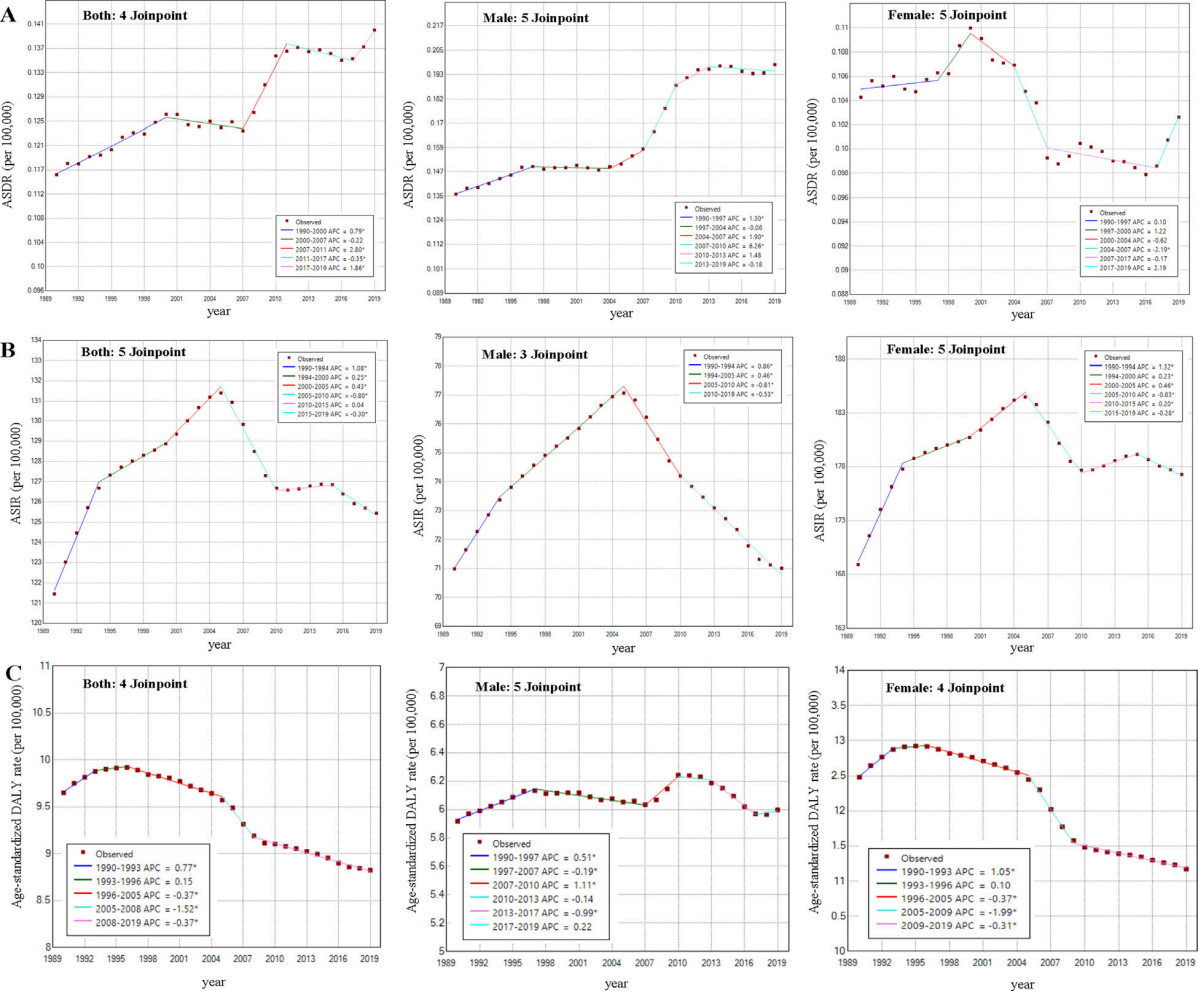
Joinpoint regression analysis of age-standardized death rate, age-standardized incidence rate and age-standardized disability-adjusted life years rate due to peripheral arterial disease recorded (China, 1990–2019) (Depicted as annual percentage change for each time segment using the slope of the graph) **(A)** Joinpoint regression analysis of age-standardized death rate for both sexes (5 time segments; 4 joinpoints), male (6 time segments; 5 joinpoints) and female (6 time segments; 5 joinpoints), respectively. **(B)** Joinpoint regression analysis of age-standardized incidence rate for both sexes (6 time segments; 5 joinpoints), male (4 time segments; 3 joinpoints) and female (6 time segments; 5 joinpoints), respectively. **(C)** Joinpoint regression analysis of age-standardized disability-adjusted life years rate for both sexes (5 time segments; 4 joinpoints), male (6 time segments; 5 joinpoints) and female (5 time segments; 4 joinpoints), respectively. * Indicates that the annual percentage change is significantly different from zero at the alpha = 0.05 level. ASDR, age-standardized death rate; ASIR, age-standardized incidence rate.

As shown in Figure 4 of the Joinpoint regression analysis, ASDR due to PAD burden in China showed an increasing trend from 1990 to 2019, with an average annual increase of 0.66% (95% CI: 0.50%–0.81%). Among them, ASDR in male patients increased by 1.24% (95% CI: 0.96%–1.52%) per year on average, especially between 2007 and 2011, with the greatest increase, with AAPC reaching 6.26% (95% CI: 4.85%–7.70%). And the ASDR of female patients showed a trend of “rising-declining-rising” during 1990–2019, with an overall AAPC of −0.08% (95% CI: −0.40%–0.25%). In addition, the ASIR for the burden of PAD in China continued to increase until 2005, and showed a decreasing trend after 2005. The change in ASIR for male patients in 2019 from 1990 was not significant, with a mean decrease of −0.01% (95% CI: −0.04%–0.02%) per year. In contrast, female ASIR showed an upward trend with an average annual increase of 0.16% (95% CI: 0.14%–0.18%). The age-standardized DALY rate was on an upward trend from 1990 to 1996, turning downward after 1996 with an overall AAPC of −0.32% (95% CI: −0.39% to −0.24%). For females, the trend remained largely consistent with the former, with an AAPC of −0.38% (95% CI: −0.43% to −0.34%). In contrast, the mean annual change for males was not significant (Supplementary Table S3).

We explored the temporal trends in PAD crude death, incidence and DALY rates for different age groups from 1990 to 2019. Death rates in each group between the age groups of 40–59 years showed a decreasing trend from 1990 to 2019, while all subgroups aged 60 years and older showed an increasing trend. The 80–84 and 40–44 age groups had the highest and lowest death rates in the trend, with AAPCs of 0.85% (95% CI: 0.56%–1.15%) and −0.40% (95 CI: −0.61% to −0.19%), respectively. Regarding the temporal trend of incidence in the age subgroups, there was an increasing trend in the age subgroup of 74 years and younger, while the increase in the subgroup of 75 years and older was very slight, and even a decreasing trend was observed in the three groups of 75–89 years. The most significant annual increase in incidence was seen in these two groups between 45 and 54 years of age, with an AAPC of 0.22% (95% CI: 0.19%–0.25%) (Supplementary Table S4).

### Predictions of Mortality and Incidence of PAD From 2020 to 2030

**FIGURE 5:**
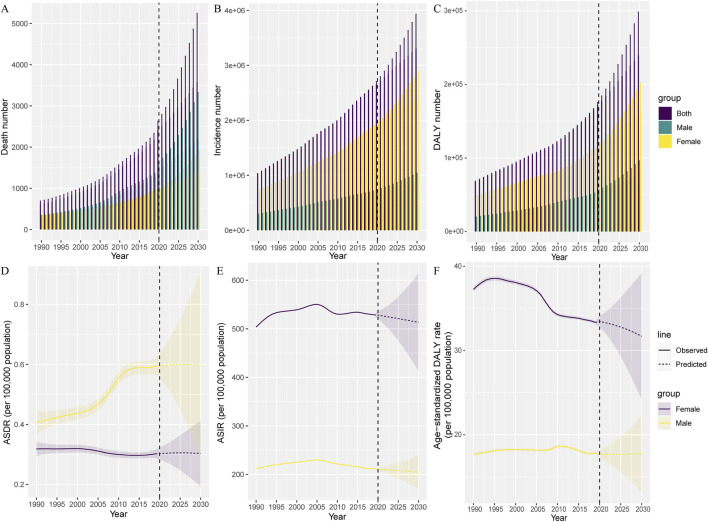
Temporal trends and forecasted curves from 2020 to 2030 for the number of deaths **(A)**, incidences **(B)** and disability-adjusted life years **(C)** and age-standardized death rate **(D)**, age-standardized incidence rate **(E)**, and age-standardized disability-adjusted life years rate **(F)** of peripheral arterial disease by sex (China, 1990–2019). The vertical dashed line indicates where the prediction starts. Solid lines and dash lines represent the observed and the predicted age-standardized death rate, age-standardized incidence rate and age-standardized disability-adjusted life years rate of peripheral arterial disease; The shading indicates the predicted distribution for the 95% uncertainty interval. ASDR, age-standardized death rate; ASIR, age-standardized incidence rate; DALY, Disability-Adjusted Life Years.

Based on GBD data of PAD from 1990 to 2019 in China, we further predicted the numbers and ASRs of mortality, incidence and DALY in the next 10 years (Figure 5). From 2020 to 2030, the number of new cases, deaths and DALYs of PAD patients in China will continue to rise. It is expected that by 2030, the number of deaths due to PAD in China will increase to 3,584 (95% UI: 1,922–5,245), the total number of new cases will increase to 3.3 million (95% UI: 2.67–3.93),and DALY will increase to 239,636 (95% UI: 181,035–298,236). The increase in the number of PAD deaths was dominated by male patients with 2,183 (95% UI: 1,038–3,328). The number of new cases and DALYs was predominantly female at 2.42 million (95% UI: 1.94–2.89) and 163,101 (95% UI: 124,372–201,829), respectively (Figures 5A, B). Both ASDR and age-standardized DALY rates will continue to decline from 2020 to 2030 for both males and females. The ASDR for males and females decreased slightly after rising to the highest point in 2026 and 2025, respectively, but overall, the ASDR in China remained on an upward trend through 2030 (Figures 5C, D). Subsequently, three age groups, 60–64, 70–74, and 80–84, were selected to forecast the number of deaths, incidences, and DALYs, as well as crude death rate, crude incidence rate, and crude DALY rate in the next 10 years. As shown in Supplementary Figure S2, there was an overall decreasing trend in the crude incidence rate for each age group from 2020 to 2030, and the decreasing trend was more pronounced in elderly male patients. Similarly, DALY also showed a decreasing trend in each age group, with a more obvious decrease in the older age groups.

## Discussion

This study provides a comparison of the burden of PAD in China with global levels by the standardized methodology used in the GBD study. From 1990 to 2019, the absolute number of new cases, prevalence, deaths, DALYs, YLDs and YLLs of PAD patients in China all increased significantly. The number of new cases, the number of diseases and YLD as a proportion of global cases increased significantly in China, while the number of deaths and DALYs as a proportion of global cases increased to a lesser extent.

After adjusting for age effects, the ASRs for each PAD burden metric in 2019 did not change significantly compared to 1990, with ASIR, ASPR, and ASDR showing an upward trend and age-standardized DALY, YLD rates showing a downward trend. In the next 10 years of projections, ASDR for Chinese PAD patients is on an upward trend, while ASIR and DALY rates are on a downward trend. The number of deaths is predominantly male, while the number of incidences and DALYs is predominantly female.

The potential reason why male patients with PAD are more likely to experience death may be that women may have a survival advantage in coronary heart disease and stroke. Population ageing and higher increases in the prevalence of non-communicable chronic diseases in men are likely to exacerbate the rise in their mortality rates [17, 18]. Several studies showed women had a lower risk of major adverse cardiovascular events and all-cause mortality at 30-month follow-up [19]. In addition, a global epidemiological study of PAD also found a higher incidence of PAD in women than in men in middle- and low-income areas [7]. These results are consistent with the trends we observed. The American Heart Association (AHA) believes it may be related to the fact that women are more aware of PAD and its associated risk factors than men and are more likely to seek medical care and make a diagnosis [20]. In contrast, we believe that this may be due to the influence of various factors such as living environment, occupation, social policy and medical level of women in low- and middle-income countries. The high prevalence of peripheral arterial disease in women may be inherent to the disease mechanism or a combination of other potential risk factors in developing countries, such as secondhand smoke, obesity and socioeconomic inequality [9, 21, 22]. Women are also affected by hormone-altering conditions such as estrogen fading and oral contraceptive use [23, 24]. In addition, there has been a decline in mortality due to peripheral arterial disease in women, which further prolongs survival, and this may be one of the reasons for the continued increase in the prevalence of peripheral arterial disease in women.

Previous studies have shown that an important risk factor for PAD is aging [1]. And China’s population is aging rapidly. It is expected that the number of people aged 70 and above in China will reach 215 million in 2050 from more than 80 million in 2010 [25]. Our results demonstrated the number of incidence and prevalence in China is mainly concentrated between 50 and 80 years of age, with the largest number of people in the 65–69 age group. The number of DALYs was concentrated between 65 and 85 years of age, while the number of deaths was mainly distributed between 75 and 90 years of age. The main cause of death in patients with PAD is the presence of major adverse cardiac and cerebrovascular event [1], which is apparently more likely in older age groups, which could also explain the higher age distribution of deaths. In the next 10 years of projection, the number of incidences, death and DALYs of PAD in China will continue to increase in all age groups.

The substantial increase in death and disability in China from 1990 to the time projected for 2030 is primarily due to population growth and aging [15]. Cardiovascular risk factors, including smoking, hypertension, diabetes mellitus, and hyperlipidemia, are more likely to be present and persistent in middle-aged and older adults. We found that smoking can cause 33.58% of PAD-related deaths in China. The effect of smoking was even more prominent in male patients, causing 49.22% of PAD-related deaths. China has the highest number of smokers in the world, with a standardized smoking rate of 26.0% over the age of 15 years, compared to 48.4% in men, and a smoking rate of about 2%–4% in Chinese women [9, 26]. However, some studies have shown that nearly 40% of non-smoking Chinese women are exposed to secondhand smoke and have a corrected odds ratio for developing PAD of 1.67. Moreover, there is a dose-response relationship between the amount and duration of secondhand smoke exposure on the increased prevalence of PAD [27]. Therefore, national policies and medical guidance should strengthen tobacco restrictions and take more effective promotional measures to reduce the burden of smoking-induced PAD as well as to reduce the effects of secondhand smoke on family members.

In the analysis of risk factors attributed to PAD, we found that hypertension and diabetes had a higher percentage of impact on disease burden in female patients. According to a recent guideline, about 244.5 million Chinese adults have hypertension, accounting for 23.2% of the population. Of these, only 15.3% have better control of their blood pressure [28]. Interestingly, the risk of PAD is associated with high systolic blood pressure, while there is usually no significant correlation with diastolic blood pressure. Therefore, we should be aware of this feature when testing blood pressure levels and performing ABI measurements [29]. In addition, numerous studies have confirmed that diabetes is closely associated with the risk of PAD. The risk of intermittent claudication is twice as high in diabetic patients as in non-diabetic patients [30], and the risk of amputation is five times higher [31]. This may be related to the susceptibility of diabetes to induce peripheral neuropathy and microangiopathy. Therefore, paying attention to PAD risk factors and controlling blood pressure and blood glucose are necessary to reduce the burden of PAD in Chinese, especially elderly female patients. A number of healthy lifestyle practices targeting risk factors for PAD have therapeutic benefits, including smoking cessation, exercise therapy, and dietary modifications.

Socioeconomics is often determined by conditions such as income, occupation, literacy and health insurance. Lower socioeconomic status and education levels are associated with a higher prevalence of cardiovascular disease, especially in low-middle-income countries [32]. Therefore, for China, there is a need to strengthen policy support for areas with relatively low levels of economic and medical care, such as optimizing the allocation of medical resources for regular support of healthcare personnel, strengthening disease awareness, promoting telemedicine services, and enhancing the capacity of primary care services. In addition, industrial lead dust pollution in the work or residential environment will significantly increase the prevalence of PAD. This may be related to inflammation, oxidative stress leading to vascular endothelial damage and dysfunction [33]. The lead exposure is strongly associated with hypertension, which also contributes to the prevalence of PAD [34]. Socioeconomic improvement will be achieved through policy support, provision of vocational training, strengthening of community health promotion, and better healthcare coverage in low-income areas. For example, governments and communities can implement measures such as free blood lead testing for susceptible populations and targeted education.

### Limitation

There are several limitations to this study. First, mortality is of very limited usefulness in describing the epidemiology of PAD because few patients die from PAD itself. Therefore, PAD may be subject to errors in the determination of mortality endpoints, affecting the assessment of burden. Second, data on PAD under 40 years of age are missing from the GBD2019 database. Considering the increasing trend towards a younger incidence of atherosclerotic vascular disease such as coronary heart disease and stroke, it is equally reasonable to suspect that PAD may be present in younger people. Third, the BAPC model is highly dependent on the accuracy and time-series of the data. The interactions between factors may form complex non-linear relationships, which need to be further analyzed. In addition, the impact of the uneven development of economic, medical and living conditions on the distribution of the burden of PAD in different regions, such as urban and rural areas, is missing.

### Conclusion

This research can help the government and healthcare providers understand the burden of PAD, especially the characteristics of the impact on different age groups, genders, and risk factors, and develop customized medical prevention and management strategies to reduce the burden of PAD. Strengthening early identification and diagnosis of PAD, especially strengthening policy and economic support in economically and medically underdeveloped areas, strengthening publicity on the prevention and treatment of PAD, actively advocating smoking and exercise cessation, controlling risk factors, and health check-ups for female patients in particular.
