# Supplementary material for: The Burden of Peripheral Artery Disease in China From 1990 to 2019 and Forecasts for 2030: Findings From the Global Burden of Disease Study 2019
Source: Int J Public Health. 2024 Dec 17;69:1607352. doi: 10.3389/ijph.2024.1607352 (PMC11685024; doi:10.3389/ijph.2024.1607352)
Supplement: Supplementary file 1 [file DataSheet1.DOCX]

Supplemental table 1 Comparison of age-standardized incidence, prevalence, death rate, DALY, YLD, and YLL rates of PAD in China and in the world, 2019.

| Variable (per 100,000) | China | World |
| --- | --- | --- |
| ASIR | 125.43(109.07-143.36) | 127.11(111.28-145.44) |
| ASPR | 1423.78(1234.84-1625.31) | 1401.85(1228.48-1589.39) |
| ASDR | 0.14(0.11-0.18) | 1.01(0.56-1.74) |
| Age-standardized DALY rate | 8.82(5.14-14.40) | 19.55(12.91-30.21) |
| Age-standardized YLD rate | 6.87(3.19-12.47) | 6.29(2.97-11.35) |
| Age-standardized YLL rate | 1.96(1.60-2.40) | 13.26(7.71-22.61) |

ASIR, age-standardized incidence rate; ASPR, age-standardized prevalence rate; ASDR, age-standardized death rate; DALY, Disability-Adjusted Life Years; YLD, Years Lived with Disability; YLL, Years of Life Lost.

Supplemental table 2. The age distribution of overall incidence, prevalence, death, DALY, YLD and YLL numbers caused by AA in 2019 and their percentage changes compared with that in 1990.

| Age group (year) | Incidence,  number(95%UI) | | Prevalence,  number(95%UI) | | Death,  number(95%UI) | | DALY,  number(95%UI) | | YLD,  number(95%UI) | | YLL,  number(95%UI) | |
| --- | --- | --- | --- | --- | --- | --- | --- | --- | --- | --- | --- | --- |
|  | 2019  (million) | Change  (%) | 2019  (million) | Change  (%) | 2019  (cases) | Change  (%) | 2019  (person-years) | Change  (%) | 2019  (person-years) | Change  (%) | 2019  (person-years) | Change (%) |
| 40-44 | 0.10(0.08-0.13) | 60.29 | 0.52(0.38-0.67) | 60.50 | 21(16-26) | 33.56 | 967(773-1201) | 33.54 | 6(1-14) | 27.84 | 961(765-1200) | 33.58 |
| 45-49 | 0.22(0.14-0.31) | 150.23 | 1.45(1.12-1.83) | 151.38 | 35(27-44) | 131.30 | 1508(1170-1925) | 130.23 | 48(9-122) | 99.70 | 1460(1133-1848) | 131.39 |
| 50-54 | 0.33(0.25-0.42) | 179.72 | 2.82(2.14-3.63) | 184.43 | 59(46-75) | 150.21 | 4281(2938-6377) | 136.04 | 2080(839-4009) | 122.68 | 2201(1717-2795) | 150.24 |
| 55-59 | 0.33(0.21-0.47) | 130.34 | 3.32(2.61-4.04) | 138.18 | 75(59-96) | 107.31 | 9461(5078-16084) | 91.70 | 7018(2798-13676) | 86.86 | 2442(1903-3104) | 107.12 |
| 60-64 | 0.37(0.27-0.48) | 132.04 | 3.84(3.00-4.67) | 142.60 | 109(87-136) | 128.07 | 17164(9114-29542) | 97.10 | 14138(6208-26278) | 91.57 | 3026(2413-3793) | 127.86 |
| 65-69 | 0.45(0.29-0.64) | 166.69 | 4.73(3.85-5.76) | 179.08 | 202(161-250) | 191.12 | 30393(16281-55505) | 130.47 | 25693(11454-51264) | 122.06 | 4700(3750-5818) | 190.64 |
| 70-74 | 0.35(0.25-0.47) | 156.81 | 4.21(3.32-5.20) | 168.93 | 290(234-351) | 205.28 | 32970(17890-56996) | 127.48 | 27433(12403-51575) | 116.41 | 5537(4465-6687) | 204.61 |
| 75-79 | 0.23(0.16-0.31) | 159.39 | 3.26(2.64-3.94) | 172.01 | 340(278-417) | 225.71 | 27903(15066-50942) | 136.56 | 22774(9892-45578) | 122.97 | 5130(4189-6295) | 224.23 |
| 80-84 | 0.15(0.10-0.20) | 232.27 | 2.50(2.05-3.02) | 249.23 | 454(369-563) | 335.45 | 23134(13273-40169) | 211.76 | 17879(7852-35408) | 188.16 | 5255(4273-6513) | 332.22 |
| 85-89 | 0.06(0.04-0.08) | 339.90 | 1.32(1.10-1.59) | 367.62 | 385(311-486) | 487.85 | 12787(7358-21235) | 325.42 | 9374(4176-17966) | 287.18 | 3414(2759-4300) | 483.68 |
| 90-94 | 0.017(0.011-0.024) | 506.27 | 0.42(0.36-0.50) | 567.69 | 179(131-257) | 593.64 | 4207(2471-7008) | 496.69 | 2974(1328-5740) | 465.75 | 1232(903-1765 | 587.42 |
| >95 | 0.003(0.002-0.005) | 619.01 | 0.09(0.08-0.11) | 712.86 | 60(40-100) | 825.34 | 955(545-1612) | 666.49 | 643 (263-1294) | 616.36 | 311(211-518) | 796.11 |

DALY, Disability-Adjusted Life Years; YLD, Years Lived with Disability; YLL, Years of Life Lost.

Supplemental table 3. Joinpoint regression analysis of ASDR, ASIR and age-standardized DALY rate by sex grouping due to PAD in China, 1990-2019

| **Sex group** | **ASDR** | | **ASIR** | | **age-standardized DALY rate** | |
| --- | --- | --- | --- | --- | --- | --- |
|  | Time range | APC (95% CI) | Time range | APC (95% CI) | Time range | APC (95% CI) |
| Both | 1990-2000 | 0.79*(0.67, 0.92) | 1990-1994 | 1.08*(1.03, 1.14) | 1990-1993 | 0.77*(0.52, 1.02) |
|  | 2000-2007 | -0.22(-0.44, 0.01) | 1994-2000 | 0.25*(0.21, 0.29) | 1993-1996 | 0.15(-0.34, 0.64) |
|  | 2007-2011 | 2.80*(2.16, 3.46) | 2000-2005 | 0.43*(0.38, 0.49) | 1996-2005 | -0.37*(-0.42, -0.31) |
|  | 2011-2017 | -0.35*(-0.65, -0.05) | 2005-2010 | -0.80*(-0.86, -0.74) | 2005-2008 | -1.52*(-1.99, -1.05) |
|  | 2017-2019 | 1.86*(0.31, 3.44) | 2010-2015 | 0.04(-0.01, 1.00) | 2008-2019 | -0.37*(-0.40, -0.33) |
|  |  |  | 2015-2019 | -0.30*(-0.36, -0.24) |  |  |
| **AAPC** | **1990-2019** | **0.66#(0.50, 0.81)** | **1990-2019** | **0.10#(0.08, 0.12)** | **1990-2019** | **-0.32#(-0.39, -0.24)** |
| Male | 1990-1997 | 1.30*(0.84, 1.76) | 1990-1994 | 0.86*(0.74, 0.97) | 1990-1997 | 0.51*(0.44, 0.58) |
|  | 1997-2000 | -0.08(-0.43, 0.27) | 1994-2005 | 0.46*(0.43, 0.49) | 1997-2007 | -0.19*(-0.23, -0.14) |
|  | 2000-2007 | 1.90*(0.33, 3.50) | 2005-2010 | -0.81*(-0.92, -0.70) | 2007-2010 | 1.11*(0.64, 1.57) |
|  | 2007-2011 | 6.26*(4.85, 7.70) | 2010-2019 | -0.53*(-0.56, -0.49) | 2010-2013 | -0.14(-0.58, 0.31) |
|  | 2011-2017 | 1.48(0.00, 2.98) |  |  | 2013-2017 | -0.99*(-1.20, -0.78) |
|  | 2017-2019 | -0.18(-0.51, 0.15) |  |  | 2017-2019 | 0.22(-0.21, 0.65) |
| **AAPC** | **1990-2019** | **1.24#(0.96, 1.52)** | **1990-2019** | **-0.01(-0.04, 0.02)** | **1990-2019** | **0.04(-0.04, 0.11)** |
| Female | 1990-1997 | 0.10(-0.20, 0.39) | 1990-1994 | 1.32*(1.25, 1.39) | 1990-1993 | 1.05*(0.88, 1.22) |
|  | 1997-2000 | 1.22(-0.70, 3.17) | 1994-2000 | 0.23*(0.18, 0.27) | 1993-1996 | 0.10(-0.24, 0.44) |
|  | 2000-2007 | -0.62(-1.55, 0.32) | 2000-2005 | 0.46*(0.39, 0.52) | 1996-2005 | -0.37*(-0.41, -0.33) |
|  | 2007-2011 | -2.19*(-4.03, -0.31) | 2005-2010 | -0.83*(-0.89, -76) | 2005-2009 | -1.99*(-2.16, -1.82) |
|  | 2011-2017 | -0.17(-0.36, 0.02) | 2010-2015 | 0.20*(0.13, 0.26) | 2009-2019 | -0.31*(-0.34, -0.28) |
|  | 2017-2019 | 2.19(-0.20, 4.65) | 2015-2019 | -0.28*(-0.34, -0.21) |  |  |
| **AAPC** | **1990-2019** | **-0.08(-0.40, 0.25)** | **1990-2019** | **0.16#(0.14, 0.18)** | **1990-2019** | **-0.38#(-0.43, -0.34)** |

* Indicates that the APC is significantly different from zero at the alpha=0.05 level.

#Indicates that the AAPC is significantly different from zero at the alpha= 0.05 level.

APC, annual percent change; AAPC, average annual percent change; ASDR, age-standardized death rate; ASIR, age-standardized incidence rate; DALY, Disability-Adjusted Life Years.

Supplemental table 4. The average annual percent changes in PAD incidence and death rate by age group, 1990-2019.

| Age group (years) | Death rate | | Incidence rate | | DALY rate | | |
| --- | --- | --- | --- | --- | --- | --- | --- |
|  | AAPC | 95% CI | AAPC | 95% CI | | AAPC | 95% CI |
| 40-44 | -0.40# | -0.61, -0.19 | 0.21# | 0.17, 0.25 | | -0.40# | -0.60, -0.20 |
| 45-49 | -0.11 | -0.64, -0.43 | 0.22# | 0.19, 0.25 | | -0.13 | -0.64, 0.39 |
| 50-54 | -0.16 | -0.41, 0.08 | 0.22# | 0.19, 0.25 | | -0.37# | -0.51, -0.23 |
| 55-59 | -0.26 | -0.56, 0.05 | 0.18# | 0.12, 0.25 | | -0.45# | -0.60, -0.31 |
| 60-64 | 0.03 | -0.19, 0.25 | 0.15# | 0.12, 0.18 | | -0.41# | -0.45, -0.37 |
| 65-69 | 0.41# | 0.20, 0.63 | 0.11# | 0.08, 0.14 | | -0.39# | -0.44, -0.34 |
| 70-74 | 0.69# | 0.47, 0.91 | 0.03 | 0.00, 0.07 | | -0.38# | -0.42, -0.33 |
| 75-79 | 0.74# | 0.49, 0.99 | -0.04 | -0.08, 0.00 | | -0.35# | -0.40, -0.30 |
| 80-84 | 0.85# | 0.56, 1.15 | -0.06# | -0.09, -0.04 | | -0.29# | -0.40, -0.18 |
| 85-89 | 0.91# | 0.51, 1.31 | -0.03# | -0.04, -0.02 | | -0.16# | -0.24, -0.07 |
| 90-94 | 0.50# | 0.22, 0.78 | 0.02 | -0.01, 0.05 | | -0.04 | -0.13, 0.04 |
| >95 | 0.81# | 0.59, 1.03 | 0.01 | -0.03, 0.06 | | 0.23# | 0.08, 0.37 |

#Indicates that the AAPC is significantly different from zero at the alpha= 0.05 level.

AAPC, average annual percent change; DALY, Disability-Adjusted Life Years.

Supplemental figure 1.


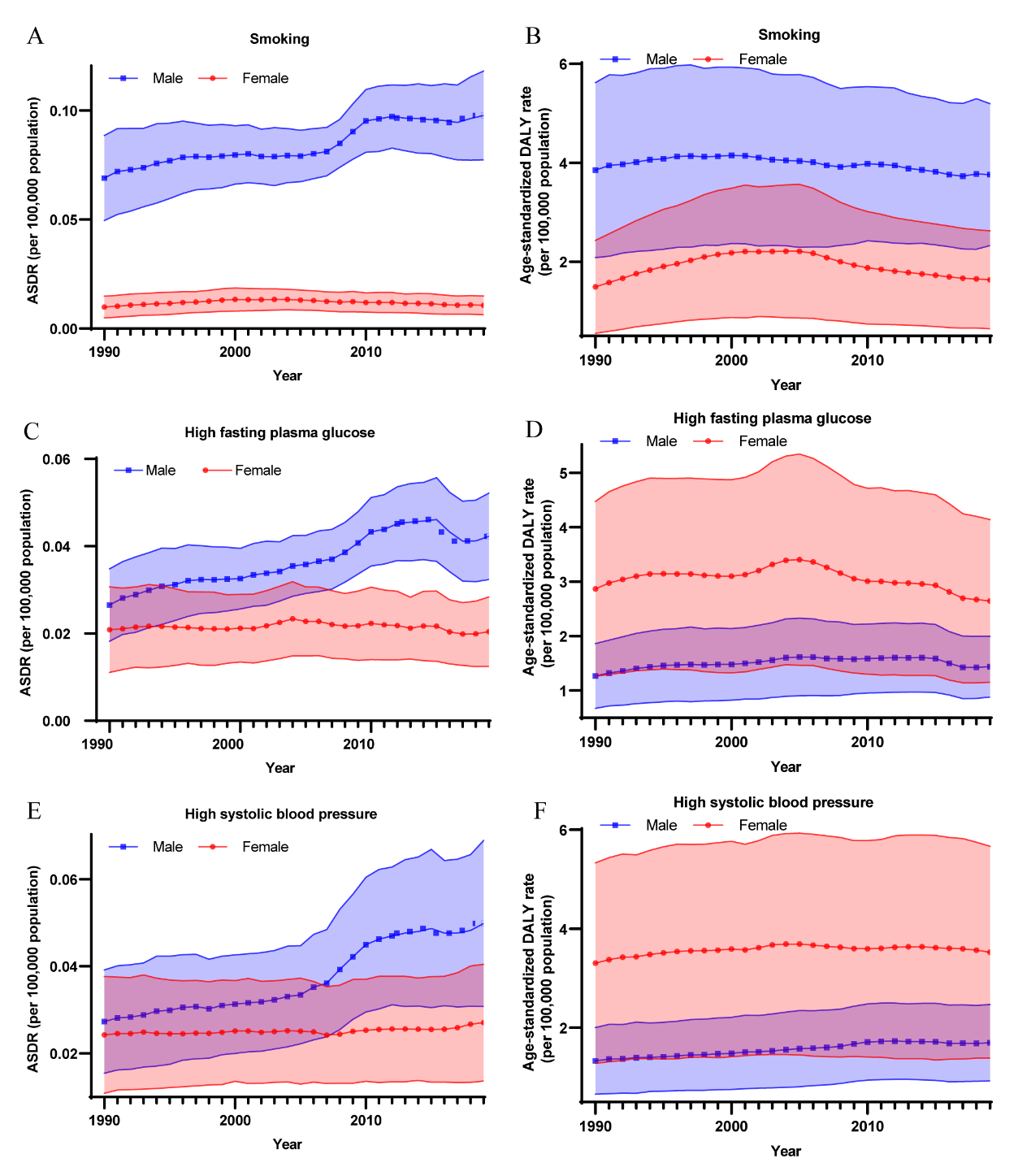


Supplemental figure 1. Annual change in ASDR and age-standardized DALY rates from 1990 to 2019 due to major risk factors for PAD burden. Smoking causes annual changes in ASDR (A) and age-standardized DALY rate (B). High fasting plasma glucose causes annual changes in ASDR (C) and age-standardized DALY rate (D). High systolic blood pressure causes annual changes in ASDR (E) and age-standardized DALY rate (F). ASDR, age-standardized death rate; DALY, Disability-Adjusted Life Years.


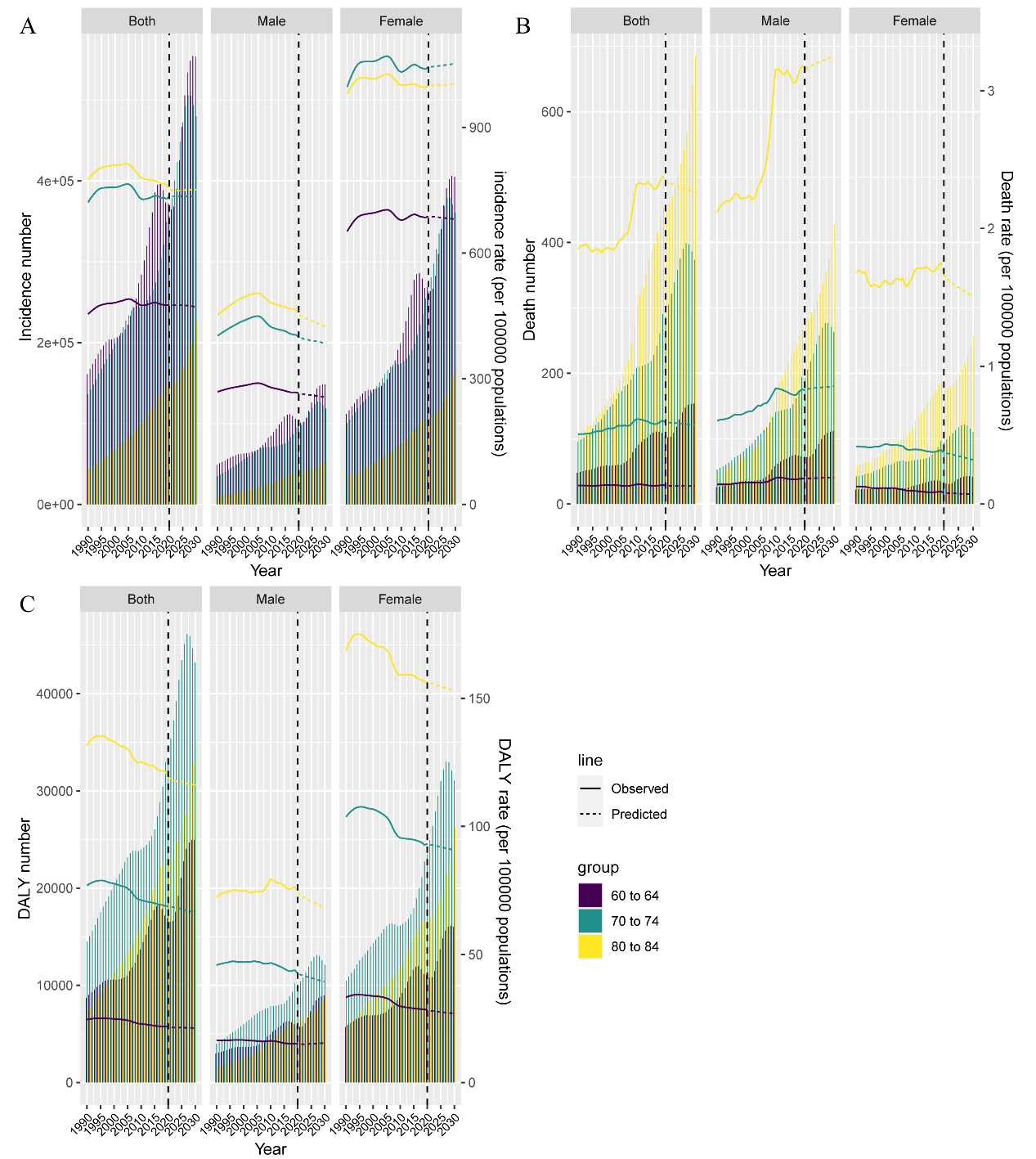


Supplemental figure 2. Temporal trends and forecasted the number of incidences, deaths and DALYs by age group, as well as crude incidence, death and DALY rates of PAD, from 2020 to 2030 in China. (A) Predicted number of incidence and crude incidence rates in the 60-64, 70-74, and 80-84 age groups. (B) Predicted number of deaths and crude death rates in the 60-64, 70-74, and 80-84 age groups. (C) Predicted number of DALYs and crude DALY rates in the 60-64, 70-74, and 80-84 age groups. The vertical dashed line indicates where the prediction starts. Solid lines and dash lines represent the observed and the predicted crude death rates and crude incidence rates of PAD. ASDR, age-standardized death rate; ASIR, age-standardized incidence rate.
